# Supplementary material for: Cryptic developmental events determine medulloblastoma radiosensitivity and cellular heterogeneity without altering transcriptomic profile
Source: Commun Biol. 2021 May 21;4:616. doi: 10.1038/s42003-021-02099-w (PMC8139976; doi:10.1038/s42003-021-02099-w)
Supplement: Supplementary file 2 — Supplementary Information [file 42003_2021_2099_MOESM2_ESM.pdf]

Supplementary Figures for:

Cryptic developmental events determine medulloblastoma radiosensitivity and cellular heterogeneity without altering transcriptomic profile.

Daniel Shiloh Malawsky**[1], Seth J. Weir**[1]Jennifer Ocasio[1,2], Benjamin Babcock[1], Taylor Dismuke[1], Abigail H. Cleveland[1,3], Andrew M. Donson[4,5], Rajeev Vibhakar[4,5], Kirk Wilhelmsen[1,6,7], Timothy R. Gershon*[1,2,8]

* corresponding author
** contributed equally to the work

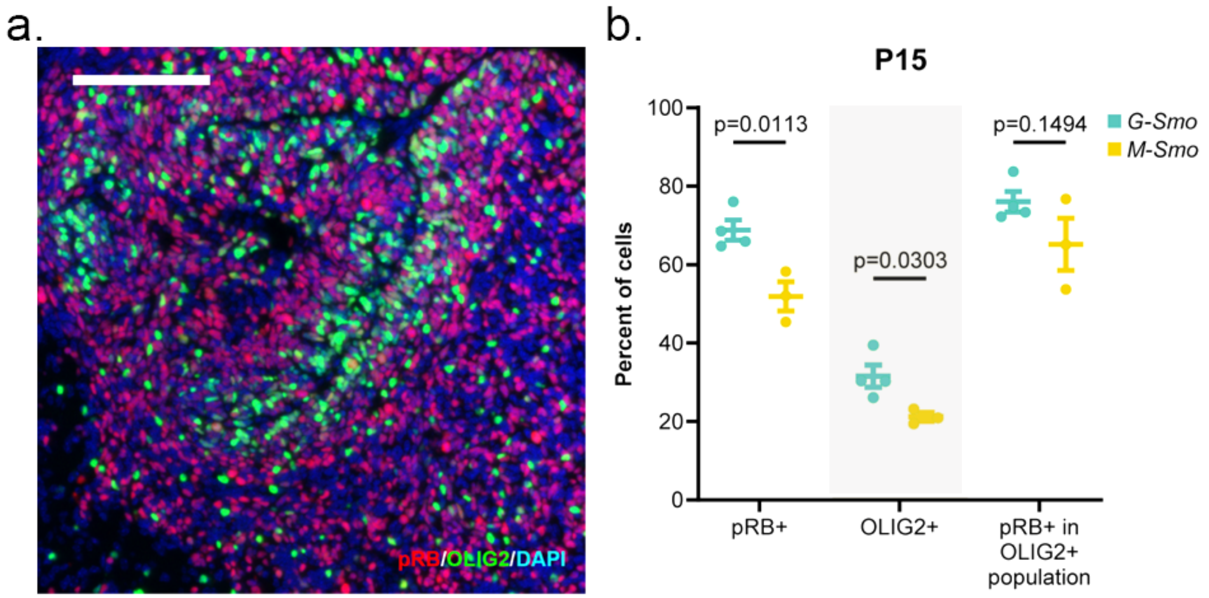

Supplementary Fig. 1 Proliferation is similar in OLIG2+ subsets of *G-Smo* and *M-Smo* tumors. (A) IHC for pRB and OLIG2 in a representative *G-Smo* tumor, including a perivascular region where OLIG2+ cells are more frequent. Scale bar = 100 µm. (B) Quantification of the fractions of pRB+ cells and OLIG2+ cells in *G-Smo* and *M-Smo* tumors, and the fraction of OLIG2+ cells that are pRB+. *G-Smo* tumors show significantly more pRB+ cells and OLIG2+ cells, but the pRB+ fraction of OLIG2+ cells is similar in both genotypes. Points represent values for individual replicates, bars indicate the means and whiskers indicate the SEM.

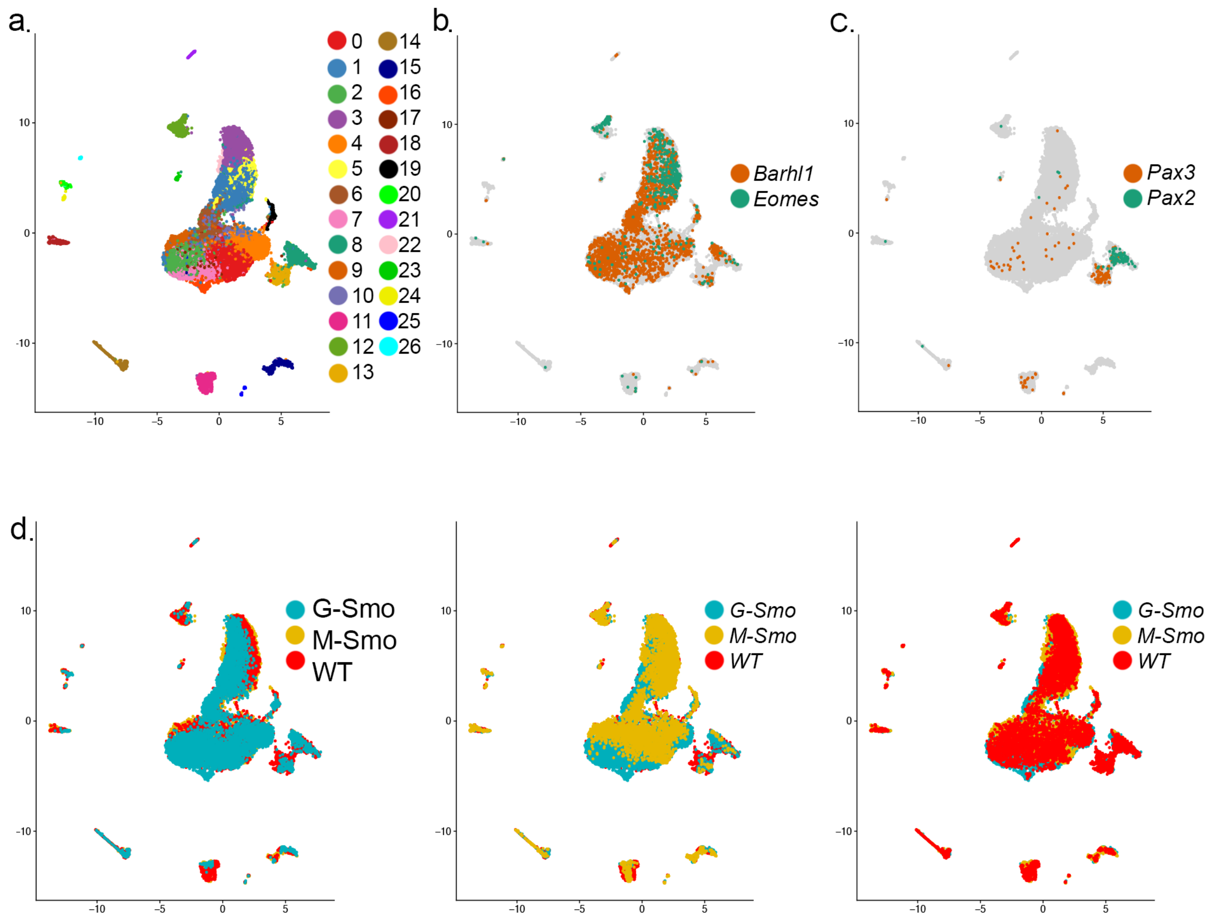

Supplementary Fig. 2 Harmony co-clusters medulloblastoma cells with P7 WT CGNPs and stromal cells from tumors and WT cerebella. (A) UMAP plot of HARMONY analysis of *G-Smo*, *M-Smo* and P7 WT cerebella, color-coded by cluster. (B) Feature plot of CGNP marker *Barhl1* and UBC marker *Eomes*, identifying the *Atoh1* lineage. (C) Feature plot of cerebellar interneuron markers *Pax3* and *Pax2* identifying the interneurons of ventricular zone lineage. (D) UMAPs as in (A), with genotype color-coded. The UMAP is reiterated with each genotype plotted on top in a separate iteration, to prevent over-plotting from obscuring each genotype.
