# Supplementary material for: Cryptic developmental events determine medulloblastoma radiosensitivity and cellular heterogeneity without altering transcriptomic profile
Source: Commun Biol. 2021 May 21;4:616. doi: 10.1038/s42003-021-02099-w (PMC8139976; doi:10.1038/s42003-021-02099-w)
Supplement: Supplementary file 3 — Description of Additional Supplementary Files [file 42003_2021_2099_MOESM3_ESM.pdf]

Description of Additional Supplementary Files

File name: Supplemental Data 1
Description: Microarray comparison of *G-Smo* vs *M-Smo* tumors.

File name: Supplemental Data 2
Description: Microarray comparison of *G-Smo* vs *M-Smo* tumors in Schuller et al. 2008.

File name: Supplemental Data 3
Description: Microarray comparison of *G-Smo* vs *M-Smo* tumors, untreated vs 2 hours after radiation.

File name: Supplemental Data 4
Description: Cluster markers in *G-Smo* vs *M-Smo* scRNA-seq data.

File name: Supplemental Data 5
Description: Genes differential in *G-Smo* clusters 1,2,7 vs *M-Smo* in scRNA-seq data.

File name: Supplemental Data 6
Description: Cluster markers in *G-Smo*, *M-Smo* & WT scRNA-seq data.

File name: Supplemental Data 7
Description: Endothelial cluster markers in *G-Smo*, *M-Smo* & WT scRNA-seq data.

File name: Supplemental Data 8
Description: Myeloid cluster markers in *G-Smo*, *M-Smo* & WT scRNA-seq data.

File name: Supplemental Data 9
Description: Fibroblast cluster markers in *G-Smo*, *M-Smo* & WT scRNA-seq data.
