# Supplementary material for: Cryptic developmental events determine medulloblastoma radiosensitivity and cellular heterogeneity without altering transcriptomic profile
Source: Commun Biol. 2021 May 21;4:616. doi: 10.1038/s42003-021-02099-w (PMC8139976; doi:10.1038/s42003-021-02099-w)
Supplement: Supplementary file 13 — Reporting Summary [file 42003_2021_2099_MOESM13_ESM.pdf]

nature research

Corresponding author(s): Timothy Gershon, MD, PhD

Last updated by author(s): 2021/03/31

Reporting Summary

Nature Research wishes to improve the reproducibility of the work that we publish. This form provides structure for consistency and transparency in reporting. For further information on Nature Research policies, see our Editorial Policies and the Editorial Policy Checklist.

Statistics

For all statistical analyses, confirm that the following items are present in the figure legend, table legend, main text, or Methods section.

n/a	Confirmed	
☐	☒	The exact sample size (*n*) for each experimental group/condition, given as a discrete number and unit of measurement
☐	☒	A statement on whether measurements were taken from distinct samples or whether the same sample was measured repeatedly
☐	☒	The statistical test(s) used AND whether they are one- or two-sided<br>*Only common tests should be described solely by name; describe more complex techniques in the Methods section.*
☐	☒	A description of all covariates tested
☐	☒	A description of any assumptions or corrections, such as tests of normality and adjustment for multiple comparisons
☐	☒	A full description of the statistical parameters including central tendency (e.g. means) or other basic estimates (e.g. regression coefficient) AND variation (e.g. standard deviation) or associated estimates of uncertainty (e.g. confidence intervals)
☐	☒	For null hypothesis testing, the test statistic (e.g. *F*, *t*, *r*) with confidence intervals, effect sizes, degrees of freedom and *P* value noted<br>*Give P values as exact values whenever suitable.*
☒	☐	For Bayesian analysis, information on the choice of priors and Markov chain Monte Carlo settings
☐	☒	For hierarchical and complex designs, identification of the appropriate level for tests and full reporting of outcomes
☒	☐	Estimates of effect sizes (e.g. Cohen's *d*, Pearson's *r*), indicating how they were calculated

Our web collection on statistics for biologists contains articles on many of the points above.

Software and code

Policy information about availability of computer code

Data collection	n/a
Data analysis	Data was processed according to the Drop-Seq Core computation protocol version 1.0.1 (http://mccarrolllab.org/wp-content/uploads/2015/05/Drop-seqAlignmentCookbook_v1.1Aug2015.pdf). Alignment was done with the GRCm38 assembly using STAR 2.7.0e. All analyses were carried out using Seurat version 3.1.1 and Seurat's implementation of SCTransform and Harmony as described with citations in the Methods. Code was run in R 3.5.0. All code is available at https://github.com/malawsky/Gershon_single-cell.

For manuscripts utilizing custom algorithms or software that are central to the research but not yet described in published literature, software must be made available to editors and reviewers. We strongly encourage code deposition in a community repository (e.g. GitHub). See the Nature Research guidelines for submitting code & software for further information.

Data

Policy information about availability of data

All manuscripts must include a data availability statement. This statement should provide the following information, where applicable:
- Accession codes, unique identifiers, or web links for publicly available datasets
- A list of figures that have associated raw data
- A description of any restrictions on data availability

The microarray data was deposited Gene Expression Omnibus database under the accession code GSE155471.. The scRNA-seq data were deposited in the Gene Expression Omnibus database under the accession code GSE155471. Expression data is also available through our web-based application: https://gsmovmsoviewer.shinyapps.is/GvMviewer.

nature research | reporting summary

April 2020

Field-specific reporting

Please select the one below that is the best fit for your research. If you are not sure, read the appropriate sections before making your selection.

☒ Life sciences ☐ Behavioural & social sciences ☐ Ecological, evolutionary & environmental sciences

For a reference copy of the document with all sections, see nature.com/documents/nr-reporting-summary-flat.pdf

Life sciences study design

All studies must disclose on these points even when the disclosure is negative.

Sample size	Sample size was determined to be adequate to show condition-specific differences in prior published studies.
Data exclusions	No data were excluded from analysis, except scRNA-seq data from cells that failed to pass specific QC measures determined prospectively and described in the methods.
Replication	Experimental findings were replicated as described in the text. Microarray findings were replicated in a published dataset. Radiation studies were performed on the specified number of replicates. Specific scRNA-seq findings were confirmed using immunohistochemistry.
Randomization	Male and female mice were randomly assigned to each treatment group.
Blinding	The technician was blinded to the sex of the mice when assigning to radiation or control groups. Unsupervised methods used to perform cluster analysis were blinded as to the genotypes of the mice.

Reporting for specific materials, systems and methods

We require information from authors about some types of materials, experimental systems and methods used in many studies. Here, indicate whether each material, system or method listed is relevant to your study. If you are not sure if a list item applies to your research, read the appropriate section before selecting a response.

Materials & experimental systems

n/a	Involved in the study
☐	☒ Antibodies
☒	☐ Eukaryotic cell lines
☒	☐ Palaeontology and archaeology
☐	☒ Animals and other organisms
☐	☒ Human research participants
☒	☐ Clinical data
☒	☐ Dual use research of concern

Methods

n/a	Involved in the study
☒	☐ ChIP-seq
☒	☐ Flow cytometry
☒	☐ MRI-based neuroimaging

Antibodies

Antibodies used	*Describe all antibodies used in the study; as applicable, provide supplier name, catalog number, clone name, and lot number.*
Validation	All antibodies were validated by the vendor. IBA1 and H2-Ea were validated further using mouse spleen as a positive control. SOX10 and OLIG2 antibodies were validated further using mouse brain sections as positive controls.

Animals and other organisms

Policy information about studies involving animals; ARRIVE guidelines recommended for reporting animal research

Laboratory animals	Mus musculus, stain C5BL/6 age and genotype as described in the text.
Wild animals	none
Field-collected samples	none
Ethics oversight	All studies were carried out under the supervision of the University of North Carolina IACAC, under protocol number 19-098.

Note that full information on the approval of the study protocol must also be provided in the manuscript.

Human research participants

Policy information about studies involving human research participants

Population characteristics	Human medulloblastomas were collected from patients presenting for surgical resection. Only patients with medulloblastoma, diagnosed by neuropathology were included, and no consenting patients with medulloblastoma were excluded.
Recruitment	No participants were recruited.
Ethics oversight	Human tumor banking was conducted under University of Colorado IRB protocol COMIRB 95-500. All material was de-identified and stripped of PHI prior to use in these studies.

Note that full information on the approval of the study protocol must also be provided in the manuscript.
